# Face to Face: The Key to Connection and Volunteer Satisfaction for LGBTQ+ Older Adults

**DOI:** 10.1093/geroni/igaf122.2401

**Published:** 2025-12-31

**Authors:** Jenna Binaco, Jillian Crocker, Hannah Friedland, Soledad Arguelles-Borge, Ashley Stripling

**Affiliations:** Nova Southeastern University, Fort Lauderdale, Florida, United States; Nova Southeastern University, Fort Lauderdale, Florida, United States; Nova Southeastern University, Fort Lauderdale, Florida, United States; Nova Southeastern University, Fort Lauderdale, Florida, United States; Nova Southeastern University, Fort Lauderdale, Florida, United States

## Abstract

Social isolation is a prominent issue for LGBTQ+ older adults, necessitating expanded efforts for support. LGBTQ+ volunteer programs are one such avenue of support, however, the growth of these programs relies on volunteer interest and satisfaction. Research on volunteer satisfaction offers valuable insight into the increased implementation of LGBTQ+ older adult volunteer programs. As such, the current study examines the satisfaction of volunteers visiting LGBTQ+ older adults. We hypothesized that volunteer satisfaction would be higher in individuals who had spent more time with their assigned participants. The sample consisted of 21 volunteers who completed a monthly report form in 2024. The form included questions regarding the duration and number of in-person and virtual interactions. Volunteer stress and satisfaction were ranked on a 4-point Likert scale. A series of Pearson correlations were conducted and results revealed a large, negative correlation (r(17)=-.744, p<.001) between volunteer stress (M = 1.74, SD = 1.05) and volunteer satisfaction (M = 3.37, SD = 1.01). The number of in-person contacts had a large, negative correlation with stress, r(17)=-.481, p=.037, and a large, positive correlation with satisfaction, r(17)=.502, p=.029. These relationships did not hold for virtual contacts, total number of contacts, or time spent with the client. The findings emphasize the significance of face-to-face interaction despite the availability and established benefits of virtual communication. The stress associated with volunteering may also reveal gaps for improving volunteer recruitment and retention. Implications include examining how volunteers engage with older LGBTQ+ adults to increase volunteer and participant satisfaction.

